# Is cognitive profiling in NF1 still optional? A systematic review of current assessment practices

**DOI:** 10.3389/fneur.2026.1794510

**Published:** 2026-04-30

**Authors:** Andrea Santangelo, Alessandra Sardi, Luca Bergonzini, Ilaria Luongo, Federica Mela, Salvatore De Pasquale, Ilaria Cecconi, Maria Stella Vari, Duccio Maria Cordelli, Maria Cristina Diana, Pasquale Striano

**Affiliations:** 1 Pediatric Neurology and Muscular Diseases Department, IRCCS G. Gaslini Institute, Genoa, Italy; 2 Department of Neurology, Rehabilitation, Ophthalmology, Genetics, Maternal and Child Health (DINOGMI), University of Genoa, Genoa, Italy; 3 IRCCS Istituto delle Scienze Neurologiche di Bologna, UOC Neuropsichiatria dell’Età Pediatrica, Bologna, Italy; 4 Department of Medical and Surgical Sciences (DIMEC), University of Bologna, Bologna, Italy; 5 International PhD College, Collegio Superiore, University of Bologna, Bologna, Italy

**Keywords:** ADHD, autism spectrum disorder, cognitive assessment, neurodevelopment, neurofibromatosis type 1, NF1

## Abstract

**Background/objectives:**

Neurofibromatosis type 1 (NF1) is a common genetic disorder often accompanied by cognitive, behavioral, and neurodevelopmental disorders. While tumor-related manifestations are well-documented, less attention has been paid to standardized assessment of neuropsychological profiles in affected children. This systematic review aimed to evaluate current assessment practices for cognitive, behavioral, and neurodevelopmental domains in pediatric NF1.

**Methods:**

A systematic search was conducted across PubMed, Scopus, Web of Science and Embase databases (April 2015–April 2025) using predefined keywords and Boolean logic. Eligibility criteria included original studies assessing neurodevelopmental, cognitive, motor, behavioral, or social–emotional outcomes in pediatric NF1 populations (0–18 years). Exclusion criteria comprised adult-only samples, reviews, non-English publications, and animal models. Study selection followed PRISMA guidelines.

**Results:**

From 414 identified records, 84 studies met inclusion criteria. A total of 35 domains were explored, with cognitive functioning (*n* = 41), executive functioning (*n* = 33), behavior (*n* = 28), and attention (*n* = 22) most frequently assessed. Over 110 assessment tools were used, including the Wechsler Intelligence Scales, BRIEF, CBCL, and Conners Rating Scales. However, 52.4% of studies focused on children aged 6 years or older, indicating that preschool-aged children remain underrepresented in the literature and limiting opportunities for early identification. Few studies employed validated diagnostic instruments for ASD or ADHD (e.g., ADOS-2, ADI-R, and K-SADS), while most relied on symptom rating scales such as the CBCL or Conners questionnaires, which assess behavioral traits but do not establish a clinical diagnosis.

**Conclusion:**

Current assessment practices in pediatric NF1 are heterogeneous and often neglect early developmental windows. The integration of standardized, age-appropriate tools and early neurodevelopmental screening—supported by interdisciplinary teams including neuropsychomotor therapists—may improve detection and management of NF1-related neurocognitive phenotypes. A shared framework for assessment is essential to enhance clinical care and research comparability.

**Systematic review registration:**

https://www.crd.york.ac.uk/PROSPERO/view/CRD420251119558, identifier CRD420251119558.

## 1 Introduction

Neurofibromatosis type 1 (NF1) is an autosomal dominant genetic condition with a birth incidence of approximately 1 in 3,000. Beyond its well-documented cutaneous and tumor-related manifestations, NF1 has been increasingly associated with a heightened risk of cognitive and behavioral difficulties, which affect up to 70% of children and adolescents with this condition (1). Such difficulties span multiple domains, including attention, executive function, visuospatial skills, language, and memory. Moreover, there is a disproportionately high prevalence of neurodevelopmental disorders such as ADHD, autism spectrum disorder (ASD), and specific learning disorders in this population (2, 3).

NF1 is caused by loss-of-function variants in the *NF1* gene, located on chromosome 17q11.2, which encodes neurofibromin, a Ras-GTPase–activating protein involved in the negative regulation of Ras/MAPK signaling. Disruption of this pathway results in increased cell proliferation and altered neuronal development and synaptic plasticity, which are believed to underline both tumorigenesis and cognitive dysfunction in NF1 (4). Clinically, NF1 is characterized by a wide phenotypic spectrum, including café-au-lait macules, axillary or inguinal freckling, Lisch nodules, neurofibromas, optic pathway gliomas, and skeletal anomalies. Importantly, many of these features appear early in childhood, offering a critical window for the identification of associated cognitive and developmental challenges.

Despite the clinical importance of these issues, there is considerable variability in how neurocognitive and behavioral functioning is assessed in children with NF1. Studies differ widely in the tools they employ, including standardized neuropsychological tests, parent-report questionnaires, clinical interviews, and observational measures. This lack of consistency poses challenges for both research comparability and clinical application (5). In some cases, assessments are tailored to specific research aims rather than guided by a shared clinical framework, contributing to a fragmented understanding of the cognitive and developmental phenotype associated with NF1.

Several systematic reviews and meta-analyses have quantified the magnitude of cognitive, behavioral, and neurodevelopmental difficulties associated with NF1, focusing on specific domains such as intellectual functioning, executive functions, attention-deficit/hyperactivity disorder symptoms, visuospatial abilities, and internalizing and externalizing symptoms. In parallel, expert consensus initiatives, such as the REiNS collaboration, have provided domain-specific recommendations for outcome selection in NF1 clinical trials. However, these contributions do not aim to provide an integrated, developmentally oriented overview of the neuropsychological domains most frequently assessed in pediatric NF1, nor a systematic synthesis of the assessment tools used across studies spanning infancy through adolescence. The present review was therefore designed to complement, rather than replicate, prior meta-analyses and expert recommendations by systematically mapping neuropsychological domains and corresponding assessment instruments across the pediatric NF1 literature, with particular attention to early developmental stages. Accordingly, the aim of this review is to map the neuropsychological domains investigated in pediatric NF1 and the assessment instruments used across studies, rather than to quantitatively synthesize clinical outcomes or effect sizes.

This systematic review, conducted in accordance with PRISMA guidelines, addresses the following research questions: (i) which neuropsychological domains are most frequently investigated in children and adolescents with neurofibromatosis type 1; (ii) to what extent neurodevelopmental domains, including cognitive, motor, and behavioral functioning are assessed across childhood and adolescence, including a quantitative evaluation of how many studies include children younger than 6 years of age; and (iii) which assessment instruments, distinguishing between performance-based neuropsychological tests and informant-report measures—are most frequently used to evaluate these domains. The review aims to support comparability across studies and to inform future research on developmental outcomes in the pediatric NF1 population.

## 2 Materials and methods

PubMed, Scopus, Web of Science and Embase databases were searched from April 2015 to April 2025, using an extensive number of keywords to answer the question: “What is known about Neurodevelopmental motor, cognitive, language and behavioral development in children with NF1.” A concept map was created to decide how the concepts were combined using AND and OR Boolean operators. In Scopus, Boolean operators W/0 and NEAR/ 0 were used to combine terms about ‘age’, old’ and ‘year’. The full electronic search strategies for each database, including all keywords, Boolean operators, and applied limits, are reported in Appendix A. The search strategy was iteratively refined to maximize sensitivity and ensure comprehensive retrieval of studies addressing neurodevelopmental motor, cognitive, language, and behavioral outcomes in children with NF.

Eligibility criteria were defined according to the Population–Concept–Context (PCC) framework. The population included children and adolescents with neurofibromatosis type 1 (0–18 years). The concept concerned the assessment of neuropsychological, behavioral, cognitive, motor, language, or social–emotional domains. The context included clinical or research settings in which standardized or structured assessment instruments were used.

All records retrieved from the database searches were imported into a reference management software, and duplicates were removed prior to screening. Title and abstract screening was independently performed by two reviewers. Full texts of potentially eligible studies were subsequently assessed for inclusion by the same reviewers. Any disagreements at either screening stage were resolved through discussion, and when consensus could not be reached, a third reviewer was consulted for arbitration. The study selection process followed PRISMA recommendations and is summarized in the PRISMA flow diagram.

Admissible study designs included randomized controlled trials (RCTs), cohort studies, case–control studies, and observational studies, provided they reported neuropsychological, behavioral, or neurodevelopmental assessments in children and adolescents with NF1. Reports were considered regardless of setting, although studies conducted in clinical or healthcare settings were particularly pertinent to our analysis.

Regarding report characteristics, only articles published after 2015 were included to ensure relevance and current clinical applicability. Studies had to be disseminated in English or translated into English to ensure clarity and consistency in data extraction and interpretation. Both peer-reviewed published articles and unpublished manuscripts, such as dissertations, theses, or conference abstracts, were eligible to avoid publication bias.

The same set of filters was applied across all consulted databases to ensure consistency in the search strategy. Searches were restricted to studies involving human participants, published in English, available as full-text articles, and including pediatric populations (0–18 years). Exclusion criteria further comprised animal studies, systematic reviews or narrative reviews, studies focusing exclusively on adult populations, and publications in languages other than English.

Given the focus of this review on standardized neuropsychological tools and clinically interpretable outcomes, only peer-reviewed studies were considered, as these provide a more reliable basis for comparison across studies. While the exclusion of non–peer-reviewed literature may limit the capture of very recent findings, this approach was adopted to minimize the inclusion of studies with incomplete methodological reporting or unvalidated assessment procedures.

Studies were excluded if the outcomes of interest were neither measured nor reported explicitly. Additionally, studies were not eligible if they did not present separate or clearly extractable data and tools for the outcomes specified in our review protocol.

The following data were extracted from the selected studies: population characteristics (number of participants and age range, presence of control groups where available), study design (cross-sectional or longitudinal), neuropsychological domains assessed (e.g., cognitive, motor, language, behavioral, and social–emotional), and the assessment instruments used to evaluate these domains. Frequencies of assessment instruments were calculated per domain, therefore, the same instrument could be counted multiple times within a single study if it was used to assess different neuropsychological domains.

Additionally, we included studies involving children under 6 years of age, treating as positive findings any preschool-aged cohorts identified irrespective of sample size.

The protocol for this systematic review was prospectively registered in the International Prospective Register of Systematic Reviews (PROSPERO; registration number: CRD 420251119558).

## 3 Results

An initial search of PubMed, Scopus, Web of Science and Embase, conducted between April and June 2025, yielded 414 records in total (121 from PubMed, 171 from Scopus, 64 from Web of Science and 58 from Embase). After removing 192 duplicates, 222 unique records were screened by title and abstract. Fifty-seven records were excluded at this stage because they did not meet the inclusion criteria with respect to the study population, relevance of the topic, or the domains of the outcomes.

A total of 165 full-text articles were retrieved for detailed evaluation. Of these, 81 studies were excluded due to ineligible populations (e.g., adult-only samples), use of non-English language, use of animal models, review articles, or lack of relevant outcomes of interest (e.g., motor, cognitive, language, behavioral, or social–emotional development). Ultimately, 84 studies met the inclusion criteria and were included in the qualitative synthesis. No records were excluded due to retrieval issues.

**Figure 1:**
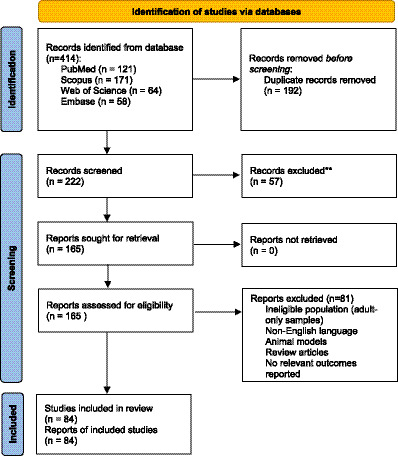
PRISMA flow diagram for the present study.

Eighty-four studies ultimately met the inclusion criteria and were included in the systematic review. No automation tools were used at any stage of screening or selection; all exclusions were performed manually by reviewers. The process is detailed in the PRISMA flow diagram (Figure 1). The main characteristics of each study are summarized in Table 1.

**Table 1:** Main characteristics of included studies.

Author; year	No. of NF1 patients	Age	Assessed domains
Allen et al. (2016) (21)	23	8–16 yrs.	Behavior, Cognitive functioning, Social Skills
Arnold et al. (2016) (22)	30	8.83 ± 1.9 yrs.	Attention, Attention and hyperactivity/impulsivity, Cognitive functioning, Language abilities, Visuospatial Skills
Arnold et al. (2021) (23)	60	8.75 ± 1.84 yrs.	Attention, Attention and hyperactivity/impulsivity, Cognitive functioning, Visuospatial Skills
Aydin et al. (2016) (24)	37	10.1 ± 3.82 yrs.	Cognitive functioning, Executive Function, Visual Skills
Barquero et al. (2015) (25)	17	10.4 ± 1.5 yrs.	ADHD symptoms, Cognitive functioning, Executive Function
Baudou et al. (2020) (26)	38	8–12 yrs.	Visuospatial Skills
Baudou et al. (2022) (27)	17	8–12 yrs.	Motor Functioning, Procedural learning and motor sequence learning
Beaussart-Corbat et al. (2021) (28)	33	3–5 yrs.	Executive Function
Begum-Ali et al. (2021) (29)	25	5–10 months	ASD, Developmental trajectiories of cognitive, Language abilities, Temperament in infancy
Bilder et al. (2016) (30)	22	N/A	ASD, ASD symptoms, Behavior
Biotteau et al. (2021) (31)	75	8–12 yrs.	Attention
Bulgheroni et al. (2019) (32)	18	6–16 yrs.	Attention and hyperactivity/impulsivity, Behavior, Cognitive functioning, Visuospatial Skills
Casnar et al. (2017) (33)	26	3–5 yrs.	Executive Function
Cesme et al. (2021) (34)	28	Mean age: 10.42	Cognitive functioning, Executive Function, Visuospatial Skills
Chisholm et al. (2022) (8)	68	3–15 yrs.	ASD symptoms, Attention and hyperactivity/impulsivity, Behavior, Cognitive functioning
Cipolletta et al.; 2018 (35)	60	6–17 yrs.	Behavior, Social Skills
Cohen et al. (2022) (36)	30	5–18 yrs.	ASD symptoms, Adaptive functioning
Coutinho et al. (2015) (37)	78	5.3–18.9 yrs.	Attention, Behavior, Cognitive functioning, Executive Function, Fine motor skills and manual dexterity, Handwriting skills (graphomotor performance), Reading ability, Visuospatial Skills
Coutinho et al. (2017) (38)	78	5–18 yrs.	Behavior, Cognitive functioning, Executive Function
Di Stasi et al. (2024) (39)	21	5–18 yrs.	Cognitive functioning
Eby et al. (2019) (40)	104	4–17 yrs.	Adaptive functioning
Eijk et al. (2018) (16)	128	2–10 yrs.	ASD, ASD symptoms, Cognitive functioning, Nonverbal cognitive ability / Nonverbal reasoning
Foy et al. (2022) (41)	71	9.8 ± 4.37 yrs.	Adaptive functioning, Behavior, Social Skills
Galasso et al. (2023) (42)	11	7–15 yrs.	Executive Function
Garg et al. (2015) (43)	36	4–16 yrs.	ASD symptoms, Cognitive functioning
Geoffray et al. (2021) (44)	48	4–18 yrs.	ASD, Behavior, Cognitive functioning
Glad et al. (2023) (45)	65	3–6 yrs.; 9–13 yrs.	Attention, Cognitive functioning, Social Skills
Glad et al. (2020) (46)	98	3–13 yrs.	Behavior, Executive Function
Guan et al. (2023) (47)	1	2 yrs.	ASD
Haas-Lude et al. (2018) (18)	14	6–12 yrs.	Attention, Cognitive functioning, Motor Functioning
Haebich et al. (2022) (48)	136	3–15 yrs.	Attention and hyperactivity/impulsivity, Executive Function, Social Skills
Haebich et al. (2023) (49)	49	4–12 yrs.	ADHD, Cognitive functioning, Nonliteral language comprehension (NLL)
Hardy et al. (2021) (50)	23	8–15 yrs.	Behavior, Cognitive functioning, Executive Function
Hernandez Del Castillo et al. (2017) (51)	23	6–10 yrs.	Cognitive functioning
Hirabaru and Matsuo (2018) (52)	760	3–15 yrs.	ADHD, ASD symptoms
Hocking et al. (2024) (53)	20	29–70 months	ASD symptoms, Behavior, Cognitive functioning, Developmental trajectiories of cognitive
Hou et al. (2020) (54)	88	6–18 yrs.	Academic skills, Attention, Cognitive functioning, Executive Function
Hou et al. (2023) (19)	88	6–18 yrs.	Attention, Behavior, Cognitive functioning, Executive Function
Hou et al. (2025) (55)	1,512	11.2 ± 3.62 yrs.	Academic skills
Huijbregts et al. (2015) (17)	15	15.3 ± 3.4 yrs.	Behavior, Executive Function, Social Skills
Iannuzzi et al. (2016) (56)	49	5–12 yrs.	Cognitive functioning, Fine motor skills and manual dexterity, Motor Functioning
Jonas et al. (2016) (57)	29	8–16 yrs.	Cognitive functioning
Kaplan et al. (2025) (58)	35	10–14 months	Interaction between caregiver and infant
Kehrer-Sawatzki et al. (2020) (59)	60	6–17 yrs.	Adaptive functioning, Behavior, Cognitive functioning
Krampe-Heni et al. (2025) (60)	32	12–47 months	Executive Function
Krvitzky et al. (2015) (61)	53	5–17 yrs.	Executive Function
Lalancette et al. (2024) (62)	36	3–19 yrs.	Cognitive functioning, Executive Function
Lalancette et al. (2023) (63)	36	4–13 yrs.	ASD symptoms, Attention
Lehtonen et al. (2015) (64)	49	11.75 ± 3.17 yrs.	Attention, Cognitive functioning, Executive Function, Nonverbal cognitive ability / Nonverbal reasoning, Visual Skills, Visuospatial Skills
Lion-François et al. (2020) (65)	32	7-13 yrs.	Attention, Executive Function
Loitfelder et al. (2015) (66)	14	12.49 ± 2.65	Behavior, Cognitive functioning, Social Skills
Lorenzo et al. (2015) (67)	39	21–40 months	Attention, Cognitive functioning, Developmental functioning in infants and toddlers, Language abilities, Temperament in infancy
Lubbers et al. (2024) (68)	278	1–18 yrs.	ASD, ASD symptoms, Behavior, Cognitive functioning, Developmental functioning in infants and toddlers, Executive Function, Nonverbal cognitive ability / Nonverbal reasoning
Maziero et al. (2019) (69)	18	8–12 yrs.	Anterograde memory, Semantic memory, Verbal working memory, Visuospatial Skills
McNeill et al. (2019) (70)	39	8–16 yrs.	ADHD, Behavior
Morotti et al. (2021) (71)	45	5–12 yrs.	Adaptive functioning, Behavior, Social Skills
Morris et al. (2021) (72)	531	2.5–83.9 yrs.	ADHD symptoms, ASD symptoms
Otthenhoff et al. (2025) (73)	31	12-16 yrs.	Attention, Executive Function, Fine motor coordination and psychomotor speed, Visual Skills
Pardej et al. (2022) (74)	41	4.95 ± 0.66 yrs.	Attention
Parmeggiani et al. (2018) (75)	36	7–11 yrs.	Attention, Behavior, Cognitive functioning, Visual Skills
Payne et al. (2019) (5)	144	6–14 yrs.	Adaptive functioning, Attention, Behavior, Executive Function, Visuospatial Skills
Payne et al. (2021) (76)	141	8–15 yrs.	Adaptive functioning, Attention and hyperactivity/impulsivity, Executive Function
Payne et al. (2016) (77)	146	11.5 ± 2.25 and 11.7 ± 1.95 yrs.	Attention, Executive Function, Visual Skills, Visuospatial Skills
Payne et al. (2020) (78)	122	3–15 yrs.	Social Skills
Vaucheret Paz et al. (2017) (94)	24	5–16 yrs.	Attention, Behavior, Cognitive functioning, Visuospatial Skills
Pierpont et al. (2018) (79)	39	8.0–16.8 yrs.	ADHD, Anxiety, Language abilities, Social Skills
Plasschaert et al. (2015) (80)	102	5–17 yrs.	ASD, ASD symptoms, Behavior
Plasschaert et al. (2016) (81)	42	8–18 yrs.	Cognitive functioning, Executive Function
Pobric et al. (2021) (82)	16	13.02 ± 1.65 yrs.	Adaptive functioning, Attention, Visual Skills, Visuospatial Skills, Working memory
Pride et al. (2023) (83)	152	3–15 yrs.	Sensory elaboration
Quental et al. (2024) (84)	1	7 yrs.	Behavior, Developmental functioning in early childhood
Remigereau et al. (2018) (85)	18	7–14 yrs.	Executive Function, Motor and ideomotor praxis skills
Rietman et al. (2018) (86)	183	6–17 yrs.	Behavior, Cognitive functioning, Physical disease severity
Rietman et al. (2017) (87)	69	4–16 yrs.	Behavior, Cognitive functioning, Motor Functioning
Riva et al. (2017) (88)	16	8–15 yrs.	Executive Function
Routier et al. (2024) (10)	47	10.0 ± 2.9 and 8.3 ± 1.8 yrs.	Cognitive functioning, Executive Function
Roy et al. (2021) (89)	40	8–12 yrs.	Executive Function, Visuospatial Skills
Slevin et al. (2024) (90)	35	5–38 months	ADHD symptoms, ASD, Adaptive functioning, Cognitive functioning
Struemph et al. (2021) (91)	55	16–31 yrs.	Adaptive functioning, Cognitive functioning
Ullrich et al. (2020) (92)	29	8–15 yrs.	Attention, Cognitive functioning, Executive Function
Van Eylen et al. (2017) (93)	39	8–18 yrs.	Attention, Behavior, Cognitive functioning, Social Skills, Visual Skills, Visuospatial Skills
Vaucheret Paz et al. (2017) (94)	24	5–16 yrs.	Attention, Behavior, Cognitive functioning, Executive Function, Visuospatial Skills
Vernet et al. (2022) (95)	42	8–12 yrs.	Reading ability, Visual Skills
Yoncheva et al. (2017) (96)	16	8–15 yrs.	Working memory

ASD, autism spectrum disorder; ADHD, attention deficit hyperactivity disorder.

**Figure 2:**
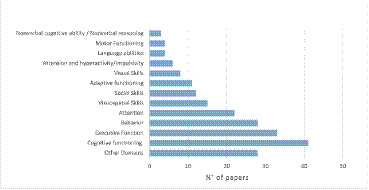
Main domains assessed in included studies.

A total of 35 cognitive and behavioral domains were investigated across the included studies. The most frequently examined domains were cognitive functioning (41 studies) executive functioning (33 studies), behavioral symptoms (28 studies), and attention (22 studies). Additional domains explored included visual and visuospatial abilities, social and language skills, and adaptive functioning (Figure 2).

Across studies, 110 distinct assessment tools were employed. The most commonly used was the Wechsler Intelligence Scale for Children (WISC), administered in 31 studies, primarily for evaluating general cognitive abilities, as well as adaptive and executive functioning, verbal and visual skills. This was followed by the Conners Rating Scales (CRS), used in 24 studies to assess attention and eventual hyperactivity symptoms, and the Behavior Rating Inventory of Executive Function (BRIEF), applied in 21 studies to evaluate everyday executive functioning, attention and behavior. Other frequently used instruments included the Child Behavior Checklist (CBCL) and the Social Responsiveness Scale (SRS).

Regarding neurodivergent conditions, diagnostic tools for autism spectrum disorder, such as the Autism Diagnostic Observation Schedule (ADOS) and the Autism Diagnostic Interview–Revised (ADI-R), were employed in 9 studies, whereas 11 assessed eventual ASD symptoms with tools such as SRS and Social Communication Questionnaire (SCQ). On the other hand, behavioral and emotional profiles were commonly explored using broad behavioral rating scales, most notably the Child Behavior Checklist (CBCL) (*n* = 16), which can also provide information on ADHD-related symptoms (*n* = 1).

Interestingly, more than half of the studies (44 papers, 52.4%) did not assess patients below the age of 6.

A comprehensive list of assessment tools and the associated domains is presented in Appendix B.

## 4 Discussion

### 4.1 Cognitive functions

Children with NF1 often exhibit broad cognitive impairments, making this one of the most common complications of the disorder. It is well-known that up to 80% of NF1 children might show deficits in one or more cognitive domains, including academic skills such as reading and math, even when general IQ remains within normal limits (6). These impairments significantly impact quality of life and academic success. Understanding the neurocognitive phenotype of NF1 is crucial for tailoring educational and clinical interventions early in development.

Cognitive functioning was most often assessed with the WISC and the Woodcock-Johnson Tests of Cognitive Abilities. Such tools could highlight some characteristic NF1 patterns, including relatively preserved verbal indices and weaker visuospatial performance (6, 7).

The cognitive profile in NF1 is hypothesized to reflect both structural brain anomalies (e.g., macrocephaly, white matter alterations) and synaptic dysfunction secondary to neurofibromin deficiency (8).

### 4.2 Executive functions

Executive dysfunction is prevalent in children with NF1 and includes difficulties with planning, working memory, inhibition, and cognitive flexibility. These deficits are independent of IQ and are often detected in both laboratory-based tasks and everyday behavior (9). Studies show that NF1 children with comorbid ADHD exhibit executive profiles similar to those with primary ADHD, but often with slower response times and more pronounced cognitive fatigue (10). Therefore, the importance of executive screening in clinical assessments appears pivotal in NF1 patients.

The most widely used instrument across studies was BRIEF, employed in 17 studies. The BRIEF’s strength lies in its ecological validity, capturing daily executive challenges from caregiver perspectives, which is particularly relevant in NF1 where traditional performance-based measures may underestimate real-world impairment (9).

Additional tools such as the Delis-Kaplan Executive Function System (D-KEFS) (*n* = 4) and the Wisconsin Card Sorting Test (WCST) (*n* = 1) were employed to assess planning, set-shifting, and abstract reasoning. Notably, recent findings show selective impairment in reactive (not spontaneous) cognitive flexibility, potentially linked to frontostriatal pathway dysfunction (11). The complementarity between ecologically-based tools and standardized executive tasks appear essential to fully capture the executive profile in NF1.

### 4.3 Behavior assessment

Behavioral challenges in NF1 frequently include emotional dysregulation, impulsivity, and social difficulties, often overlapping with symptoms of ADHD or autism spectrum disorder. These problems can persist even in the absence of global cognitive deficits, suggesting distinct neural underpinnings (12). Externalizing behaviors can significantly hinder academic progress and interpersonal relationships, reinforcing the need for behavioral screening and targeted interventions in this population (9).

Behavioral symptoms were assessed primarily using the CBCL (*n* = 16) and the Behavior Assessment System for Children (BASC) (*n* = 3). These tools offer broad coverage of internalizing, externalizing, and autistic traits—key domains in NF1, where children often exhibit co-occurring ADHD, anxiety, and social difficulties.

The BASC, in particular, adds granularity through specific indices like social adjustment and behavioral inhibition. Literature suggests that behavioral issues in NF1 stem from altered fronto-limbic circuitry and dopaminergic dysregulation (13).

### 4.4 Visuospatial skills

Visuospatial impairments are a well-recognized feature of NF1’s cognitive phenotype. Children often perform poorly on tasks involving spatial memory and visual processing, such as the Rey–Osterrieth Complex Figure or the Paired Associates Learning Task. In one study examining visuospatial performance using the Rey–Osterrieth Complex Figure Test, up to 62% of children with NF1 scored below the first percentile relative to normative data, with deficits persisting even after adjustment for IQ and attention (14). Identifying visuospatial weaknesses is essential for both clinical management and evaluating treatment efficacy in clinical trials.

These deficits have been systematically assessed using tools such as the Judgment of Line Orientation (JLO) (*n* = 5), the Beery-Buktenica Developmental Test of Visual-Motor Integration (VMI) (*n* = 1), and the Rey-Osterrieth Complex Figure Test (*n* = 6). The JLO is particularly sensitive in capturing subtle spatial orientation deficits linked to right parietal lobe dysfunction, a region frequently implicated in NF1-related structural anomalies.

The VMI, by integrating visual perception and motor coordination, helps disentangle pure perceptual issues from motor planning difficulties, both of which are relevant in NF1. Children with NF1 tend to show performance significantly below age-matched norms, often independent of IQ, suggesting a primary visuospatial processing impairment (6, 15).

Functional neuroimaging studies have linked these difficulties to hypofunction in the magnocellular visual pathway, responsible for processing low spatial frequency and high temporal resolution stimuli. Deficits in this pathway may underlie not only visual–spatial difficulties but also aspects of social cue processing, such as facial recognition and spatial attention (15).

From a clinical standpoint, these visuospatial deficits are particularly relevant for academic achievement in areas like geometry, handwriting, and reading (especially tracking and spacing), and may require targeted visual-motor integration interventions.

### 4.5 Autistic spectrum disorder

Autistic traits and clinically significant ASD symptomatology are increasingly recognized in children with NF1 (97). Several studies have used both screening instruments and diagnostic tools to characterize these features. Questionnaire-based measures such as the Social Responsiveness Scale (SRS) are primarily employed as screening instruments to quantify autistic traits and social communication difficulties along a dimensional continuum, whereas observational instruments such as the Autism Diagnostic Observation Schedule (ADOS) are considered gold-standard diagnostic tools for the direct assessment of core ASD symptoms. The combined use of these approaches allows researchers to identify both subthreshold autistic traits and clinically significant ASD presentations.

In many cases, autistic traits in NF1 appear subthreshold but remain functionally impairing—particularly in areas such as eye contact, peer relationships, and social reciprocity (16). Interestingly, these traits may reflect a distinct autism phenotype associated with NF1 rather than idiopathic ASD (97), potentially driven by abnormal Ras/MAPK pathway signaling and its downstream effects on synaptic plasticity. Moreover, some studies suggest that brain volumetric abnormalities, including increased amygdala volume and reduced gray matter in prefrontal regions, are correlated with autistic mannerisms (17).

Clinically, these findings highlight the importance of targeted screening for ASD-related symptoms in children with NF1, even in the absence of a formal diagnosis, as social difficulties may otherwise be misattributed solely to ADHD or general cognitive delays. Early identification may therefore support the implementation of interventions targeting social cognition and peer interaction.

### 4.6 Attention and attention-deficit/hyperactivity disorder (ADHD)

Attention deficits are among the most frequent neuropsychological difficulties observed in children with NF1 and represent a core component of their cognitive profile. Sustained attention impairments have been reported in over 60% of cases, with nearly 40% meeting diagnostic criteria for ADHD (6). Indeed, ADHD constitutes the most prevalent psychiatric comorbidity in this population, with reported prevalence rates ranging from 30 to 50% across studies. These attentional difficulties often interfere with academic functioning and frequently overlap with executive dysfunction.

The ADHD phenotype in NF1 is typically characterized by a predominantly inattentive presentation, often without marked hyperactivity, and commonly associated with executive deficits (9, 18). This clinical profile differs from idiopathic ADHD and has important implications for differential diagnosis and treatment planning. Neurobiologically, attention and ADHD symptoms in NF1 are thought to reflect alterations in frontostriatal circuits and dopaminergic pathways, including structural differences in the basal ganglia and prefrontal cortex (10). Importantly, executive dysfunction—particularly impairments in working memory and inhibitory control—has been shown to predict academic and behavioral outcomes beyond ADHD symptom severity alone (19).

A range of standardized instruments has been used to assess attentional functioning and ADHD-related symptoms in NF1. These include symptom-based rating scales, such as the Conners’ Rating Scales (CRS) (*n* = 2) and the ADHD DSM-IV Checklist, as well as performance-based measures of attentional processes, including the Test of Variables of Attention (TOVA) (*n* = 1), the Continuous Performance Test (CPT) (*n* = 4), and the Test of Everyday Attention for Children (TEA-Ch). These instruments capture complementary aspects of attentional functioning, including sustained attention, impulsivity, and inattention.

Among the performance-based measures identified in the literature, the Test of Everyday Attention for Children (TEA-Ch) was used in six studies to evaluate attentional functioning and in three studies to examine executive components of attention. The TEA-Ch assesses selective and sustained attention, attentional control, and aspects of cognitive flexibility through ecologically oriented tasks designed to reflect everyday attentional demands in children.

From a therapeutic perspective, children with NF1 and comorbid ADHD may benefit from methylphenidate treatment, although response patterns may differ from those observed in idiopathic ADHD (13). Early identification of attentional difficulties, together with appropriate pharmacological and behavioral interventions, may contribute to improved academic functioning and overall quality of life in this population.

### 4.7 Practical and logistical considerations in neuropsychological assessment

Across neuropsychological domains in pediatric NF1, the selection of assessment instruments is influenced not only by psychometric validity and domain coverage, but also by practical and logistical considerations. These include direct and indirect costs (e.g., licensing fees, test kits, software, and hardware requirements), examiner training and certification demands, administration and scoring time, and overall feasibility in routine practice. For example, informant-based questionnaires such as the BRIEF, CBCL, and Conners’ Rating Scales generally involve lower financial investment and minimal examiner training, making them easier to implement in large-scale screenings or resource-limited environments. In contrast, performance-based batteries such as the WISC, Woodcock–Johnson Tests of Cognitive Abilities, D-KEFS, WCST, TOVA, or the Rey–Osterrieth Complex Figure Test require specialized materials, formal training, computerized platforms in some cases, and longer administration and scoring times. Similarly, visuospatial measures like the Judgment of Line Orientation (JLO) and the Beery VMI are relatively brief and low in material burden, whereas more complex paradigms such as Paired Associates Learning tasks entail greater logistical preparation. Consequently, decisions regarding test selection in pediatric NF1 assessments often reflect a balance between methodological rigor and real-world feasibility, particularly when instruments are applied across different clinical and research settings.

### 4.8 Contribution beyond prior reviews

Several narrative reviews have previously described the neurocognitive and behavioral phenotype associated with NF1, highlighting frequent comorbidities such as symptoms related to ADHD and ASD (1, 13, 15). However, these studies were not designed to systematically map the specific neuropsychological domains most consistently affected in pediatric NF1 and the assessment instruments most commonly used to evaluate each domain. This systematic review builds on previous research by integrating evidence from 84 studies published in the last decade, providing a structured synthesis that links specific domains (e.g., attention, executive functioning, visual–spatial skills, behavior and adaptive functioning) to the tools used in practice and research. Additionally, by quantifying the proportion of studies that included children under 6 years old, this review highlights an important developmental gap in the evidence base, supporting the prioritization of earlier assessment and longitudinal research. This domain-by-tool mapping is intended to facilitate comparability across future studies and inform harmonized assessment protocols in pediatric NF1.

Compared to prior systematic reviews and meta-analyses, the present work addresses a different research objective. While meta-analytic studies have provided pooled effect estimates for specific domains in NF1, this review was not designed to quantify the magnitude of impairment, but rather to systematically map which neuropsychological domains have been investigated and which assessment instruments have been employed at different developmental stages. Accordingly, the absence of pooled effect sizes reflects a deliberate methodological choice rather than an execution limitation. Unlike domain-specific meta-analyses, this review integrates evidence from the cognitive, behavioral, motor, language and social–emotional domains within a single developmental framework. It highlights patterns in assessment practices and the heterogeneity of tools used, as well as gaps in coverage of the early childhood years. Similarly, unlike expert-based recommendations (e.g., REiNS), which use consensus-driven ratings and predefined criteria to select outcomes for clinical trials, this review uses an evidence-mapping approach based on published empirical studies. This approach avoids making judgments about instrument suitability in advance. These complementary approaches serve distinct but synergistic purposes within the NF1 research landscape.

### 4.9 Strengths, limitations and future directions

This review has several strengths. It takes a PRISMA-informed systematic approach and focuses on a decade, capturing contemporary diagnostic and assessment practices. It also provides a comprehensive synthesis of neuropsychological domains and the instruments used to assess them. Furthermore, the explicit quantification of studies including children under 6 years of age provides a clinically relevant perspective on early developmental assessment.

Nevertheless, important limitations should be acknowledged. Firstly, grey literature sources were not systematically included, which may have resulted in studies being missed despite expected overlap across databases. Secondly, preprints and other non-peer-reviewed sources were excluded in order to prioritize methodological consistency, meaning that very recent findings may be absent. Thirdly, heterogeneity across study designs, sampled age ranges, outcome definitions and instruments limited direct comparability and precluded quantitative pooling. Accordingly, we did not compute pooled effect sizes or meta-analytic estimates, and our conclusions are based on qualitative and frequency-based synthesis rather than aggregated impairment magnitude. By synthesizing assessment practices across domains and age ranges, this review establishes a basis for future harmonization efforts and the application of existing recommendations within a broader developmental context. Future work may explicitly integrate domain-level meta-analytic evidence with mapping-based syntheses to inform unified assessment frameworks across pediatric NF1 research. Future research would benefit from: (i) longitudinal studies beginning in early childhood to characterize developmental trajectories; (ii) greater harmonization of outcome measures across studies, including consensus core outcome sets; (iii) NF1-specific psychometric evaluation of commonly used tools; (iv) meta-analytic work where sufficient homogeneity exists, potentially complemented by sensitivity analyses that examine publication status and study quality.

Due to the heterogeneity of study designs and outcome measures, the present review did not compute pooled effect estimates. Therefore, statements regarding neurodevelopmental ‘risk’ should be interpreted as reflecting the consistency of findings across studies, rather than as quantitative estimates of effect size.

## 5 Conclusion

The literature consistently indicates that children with NF1 show neurodevelopmental difficulties across multiples domains. By mapping the neuropsychological domains and assessment tools used in pediatric NF1 research over the past decade, OUR work highlights the consistency of reported difficulties, the heterogeneity of assessment practices, and the limited focus on early developmental stages. These observations complement existing meta-analytic evidence and may inform future efforts toward more harmonized and developmentally sensitive outcome selection in NF1.

These outcomes reinforce the importance of comprehensive neurodevelopmental assessment in pediatric NF1 populations. Given that more than half of the studies identified in this review did not include children younger than 6 years of age, early developmental screening may represent an important opportunity to detect neurodevelopmental difficulties in children with NF1. In this context, screening instruments together with broader developmental assessments [e.g., the Bayley Scales of Infant and Toddler Development or the Griffiths III (as reported in the included studies)], may help facilitate earlier identification. Supplementary measures (e.g., the Vineland Adaptive Behavior Scales or the Child Behavior Checklist) may further support the characterization of adaptive and behavioral functioning in this age group. Early assessments not only inform educational planning but may also prevent the consolidation of secondary emotional and relational problems, particularly in children with subtle or under-recognized difficulties. In this context, multidisciplinary clinical teams with expertise in neurodevelopment may facilitate early assessment and support for children with NF1. Their contribution can help bridge the gap between diagnosis and everyday functioning, offering concrete benefits to patients and families alike. In this evolving clinical landscape, improvements in therapeutic strategies and comprehensive patient management are likely to further enhance the outcomes for individuals with NF1. Looking ahead, the future of the clinical management of neurofibromatosis type 1 appears increasingly promising. Advances in targeted therapies, such as selumetinib for treating symptomatic plexiform neurofibromas, represent an important step toward mechanism-based treatments and have already demonstrated significant clinical benefits in pediatric patients (20). At the same time, the growing focus on comprehensive, multidisciplinary management, including systematic neurodevelopmental and neuropsychological assessment, reflects a broader effort to address the full clinical complexity of NF1. Progress in therapeutic strategies and patient-centered care frameworks together suggest a more optimistic outlook for the future management of NF1.

## Data Availability

Publicly available datasets analyzed in this study are included in the article/Supplementary material, further inquiries can be directed to the corresponding author. This article is a systematic review based exclusively on previously published studies and no datasets were generated or analyzed.
