# Supplementary material for: Is cognitive profiling in NF1 still optional? A systematic review of current assessment practices
Source: Front Neurol. 2026 Apr 30;17:1794510. doi: 10.3389/fneur.2026.1794510 (PMC13173456; doi:10.3389/fneur.2026.1794510)
Supplement: Supplementary file 1 [file Supplementary_File_1.pdf]

Appendix A

((neurofibromatosis) OR (NF1)) AND (written expression)
((neurofibromatosis) OR (NF1)) AND (dyscalculia)
((neurofibromatosis) OR (NF1)) AND (intellectual disability)
((neurofibromatosis) OR (NF1)) AND (intellectual impairment)
((neurofibromatosis) OR (NF1)) AND (cognitive impairment)
((neurofibromatosis) OR (NF1)) AND (intellectual developmental disorder)
((neurofibromatosis) OR (NF1)) AND (language delay)
((neurofibromatosis) OR (NF1)) AND (language disorders)
((neurofibromatosis) OR (NF1)) AND (speech sound disorder)
((neurofibromatosis) OR (NF1)) AND (social communication disorder)
((neurofibromatosis) OR (NF1)) AND (motor disorder)
((neurofibromatosis) OR (NF1)) AND (motor abnormalities)
((neurofibromatosis) OR (NF1)) AND (developmental coordination disorder)
((neurofibromatosis) OR (NF1)) AND (sensory processing)
((neurofibromatosis) OR (NF1)) AND (visual spatial)
((neurofibromatosis) OR (NF1)) AND (visual motor)
((neurofibromatosis) OR (NF1)) AND (visual perception)
((neurofibromatosis) OR (NF1)) AND (executive dysfunction)
((neurofibromatosis) OR (NF1)) AND (executive function)
((neurofibromatosis) OR (NF1)) AND (working memory)
((neurofibromatosis) OR (NF1)) AND (problem solving)
((neurofibromatosis)OR (NF1) AND (inhibitory control)
((neurofibromatosis) OR (NF1) AND (response inhibition)
((neurofibromatosis) OR (NF1)) AND (neurodevelopmental disorders)
((neurofibromatosis) OR (NF1)) AND (neuropsychiatric assessment)
((neurofibromatosis) OR (NF1)) AND (neuropsychiatric evaluation)
((neurofibromatosis) OR (NF1)) AND (psychiatric disorders)
((neurofibromatosis) OR (NF1)) AND (neuropsychiatric disorders)
((neurofibromatosis) OR (NF1)) AND (psychomotor development assessment)
((neurofibromatosis) OR (NF1)) AND (psychometric assessments)
((neurofibromatosis) OR (NF1)) AND (neuropsychological profile)
((neurofibromatosis) OR (NF1)) AND (psychomotor delay)
((neurofibromatosis) OR (NF1)) AND (delayed psychomotor development)
((neurofibromatosis) OR (NF1)) AND (cognitive development)
(((neurofibromatosis) OR (NF1)) AND (global developmental delay))
((neurofibromatosis) OR (NF1)) AND (ADHD)
((neurofibromatosis) OR (NF1)) AND (Attention-Deficit/Hyperactivity Disorder)
(((neurofibromatosis) OR (NF1))) AND (ASD)
(((neurofibromatosis) OR (NF1))) AND (autism spectrum disorder)
(((neurofibromatosis) OR (NF1))) AND (reading)
(((neurofibromatosis) OR (NF1))) AND (dyslexia)

Appendix B

Test	Articles
**Academic skills**	
Woodcock-Johnson Test of Cognitive Abilities – III (WJ-III)	2
**Adaptive functioning**	
Adaptive Behavior Assessment System (ABAS)	1
Behavior Assessment System for Children, Second Edition (BASC-II)	2
Scales of Independent Behavior – Revised (SIB-R)	1
Vineland Adaptive Behavior Scales (VABS)	7
Wechsler Intelligence Scale for Children (WISC)	1
**ADHD**	
ADHD Rating Scale (ADHD-RS)	4
**ADHD symptoms**	
Child Behavior Checklist (CBCL)	1
Conners Rating Scales (CRS)	2
**Anterograde memory**	
Batterie d'évaluation multidimensionnelle 144 (BEM-144)	1
**Anxiety**	
Background History Form: Ademographic formwas	1
Behavior Assessment System for Children, Second Edition (BASC-II)	1
**ASD**	
Autism Behavior Checklist (ABC)	1
Autism Diagnostic Interview-Revised (ADI-R)	2
Autism Diagnostic Observation Schedule (ADOS)	8
Autism Observation Score for Infants	1
Brief Observation of Symptoms of Autism – Minimally Verbal (BOSA-MV)	1
Childhood Autism Rating Scale (CARS)	1
DSM-IV-TR criteria	1
Social Responsiveness Scale	1
**ASD symptoms**	
Autism Diagnostic Interview-Revised (ADI-R)	1
Autism Diagnostic Observation Schedule (ADOS)	2
Social Communication Questionnaire (SCQ)	1
Social Responsiveness Scale	10
**Attention**	
Attentional Network Task – Spatial Attention (ANT-SA)	1
Behavior Rating Inventory of Executive Function (BRIEF)	1
Bells Test	2
Child Behavior Checklist (CBCL)	1
Conners Rating Scales (CRS)	9
Continuous Performance Test	4
Kiddie Disruptive Behavior Disorder Schedule	1
Developmental Neuropsychological Assessment Second Edition	3

(NEPSY-II)	
Test of Everyday Attention for Children (TEA-Ch)	6
Test of Variables of Attention (T.O.V.A.)	1
Visual Search Task	1
**Attention and hyperactivity/impulsivity**	
Conners Rating Scales (CRS)	6
**Behavior**	
Alouette Test	1
Behavior Assessment System for Children, Second Edition (BASC-II)	3
Behavior Rating Inventory of Executive Function (BRIEF)	2
Child and Adolescent Disruptive Behavior Inventory (CADS)	1
Child Behavior Checklist (CBCL)	16
Children's Social Behavior Questionnaire (CSBQ)	1
Cognitive Self-Regulation Scale: Restricted and Repetitive Behaviors (CSS-RRB)	1
Conners Rating Scales (CRS)	5
Continuous Performance Test	1
Independent Behavior—Revised	1
Restricted and Repetitive Behaviors and Interests (RRBIs)	1
Strengths and Difficulties Questionnaire (SDQ)	1
Teacher Report Form (TRF)	1
Vineland Adaptive Behavior Scales (VABS)	1
YSR (Youth Self-Report – adolescent version)	1
Dysexecutive Questionnaire	1
**Cognitive functioning**	
Differential Ability Scales – Second Edition (DAS-II)	1
Kaufman Assessment Battery for Children (K-ABC)	1
Leiter International Performance Scale – Revised (Leiter-R)	1
Mullen Scales of Early Learning (MSEL)	1
Wechsler Abbreviated Scale of Intelligence (WASI)	8
Wechsler Adult Intelligence Scale (WAIS)	3
Wechsler Intelligence Scale for Children (WISC)	31
Wechsler Preschool and Primary Scale of Intelligence (WPPSI)	5
Woodcock-Johnson Test of Cognitive Abilities – III (WJ-III)	2
Woodcock-Johnson Test of Cognitive Abilities – Revised (WJ-R)	1
Dysexecutive Questionnaire	1
**Developmental functioning in early childhood**	
Griffiths III	1
**Developmental functioning in infants and toddlers**	
Bayley Scales of Infant and Toddler Development, Third Edition (Bayley III)	1
Bayley Scales of Infant Development, Second Edition (BSID-II)	1
**Developmental trajectiories of cognitive**	
Mullen Scales of Early Learning (MSEL)	2

**Executive Function**	
Behavior Rating Inventory of Executive Function (BRIEF)	17
Behavioral Assessment of the Dysexecutive Syndrome for Children (BADS-C)	1
Cambridge Neuropsychological Test Automated Battery (CANTAB)	4
Cognitive Self-Regulation Scale: Self-Assessment (CSS-SA)	1
Conners Rating Scales (CRS)	3
Continuous Performance Test	4
Controlled Oral Word Association Test (COWAT)	2
Judgment of Line Orientation (JLO)	4
Mise en situation de Fonctionnement Exécutif en Développement (MFED 1–4)	1
Modified Card Sorting Test (MCST)	1
Developmental Neuropsychological Assessment Second Edition (NEPSY-II)	2
Number Sequence Test	1
Stroop Test	5
Test for Attentional Performance in Children (KiTAP)	1
Test of Everyday Attention for Children (TEA-Ch)	3
Tower of London (TOL)	2
Delis-Kaplan Executive Function System (D-KEFS)	4
Trail Making Test Part B (TMT-B or T2B)	1
Wechsler Intelligence Scale for Children (WISC)	4
Wisconsin Card Sorting Test (WCST)	1
Dysexecutive Questionnaire	1
**Fine motor coordination and psychomotor speed**	
Grooved Pegboard Test	1
**Fine motor skills and manual dexterity**	
Purdue Pegboard test	2
**Handwriting skills (graphomotor performance)**	
Brief Handwriting Knowledge (BHK)	1
**Interaction between caregiver and infant**	
Manchester scale	1
**Language abilities**	
MacArthur Bates Communicative Development Inventory	2
Developmental Neuropsychological Assessment Second Edition (NEPSY-II)	1
The Children’s Communication Checklist, Second Edition (CCC-2)	1
Castles and Coltheart 2 Reading Test	1
Test of Word Reading Efficiency	1
**Motor and ideomotor praxis skills**	
Finger Sequencing Motor Task (Finger SM)	1
Infant Motor Profile (IMP)	1
Manual SM	1

Motricity Profile of the Child (MPO)	1
Pantomime production	1
Rey-Osterrieth Complex Figure Test	1
**Motor Functioning**	
Movement Assessment Battery for Children (M-ABC)	4
**Nonliteral language comprehension (NLL)**	
Custom-designed NLL task (sarcasm, metaphor, simile, literal language)	1
**Nonverbal cognitive ability / Nonverbal reasoning**	
Snijders-Oomen Nonverbal Intelligence Test (SON)	1
Wechsler Intelligence Scale for Children (WISC)	1
Wechsler Nonverbal Scale of Ability (WNV)	1
Spatial Working Memory Task	1
**Physical disease severity**	
Riccardi Scale modified	1
**Procedural learning and motor sequence learning**	
Visuo-Spatial Serial Reaction Time Task (SRTT)	1
**Reading ability**	
Alouette Test	2
**Reading comprehnsion**	
Battery for the Assessment of Reading and Spelling (ORLEC Lobrot)	1
**Semantic memory**	
Picture Naming Test Den30	1
Wechsler Intelligence Scale for Children (WISC)	1
**Sensory elaboration**	
Sensory profile-2	1
**Social Skills**	
Adaptive Behavior Assessment System (ABAS)	1
Autoadministered Psychiatric scales for children and adolescents	1
Diagnostic Analysis of Nonverbal Accuracy, Second Edition (DANVA-2)	1
Human figure drawing test	1
Repetitive Behavior Scale – Revised	1
Social Communication Index (SCI)	1
Social Communication Questionnaire (SCQ)	1
Social Responsiveness Scale (SRS)	5
Social Skills Improvement System (SSIS)	4
Social Skills Rating System	2
Social-Emotional Assets and Resilience Scales (SEARS)	1
**Temperament in infancy**	
Infant Behaviour Questionnaire-Revised,	1
TABS Temperament and Atypical Behavior Scale	1
**Verbal working memory**	
Wechsler Intelligence Scale for Children (WISC)	1
**Visual Skills**	
Beery-Buktenica Developmental Test of Visual-Motor Integration (VMI)	1

Cambridge Neuropsychological Test Automated Battery (CANTAB) PAL (Paired Associates Learning)	2
Coherent Motion Task	1
Developmental Eye Movement test (DEM-test	1
Fragmented Object Outlines Task	1
Motor-Free Visual Perception Test (MVPT)	1
Visuo-motor integration test	1
Wechsler Intelligence Scale for Children (WISC)	1
Bender Visual-Motor Gestalt Test	1
Corsi Blocks Task	1
**Visuospatial Skills**	
Block-Tapping Test (Wechsler's version)	1
Cambridge Neuropsychological Test Automated Battery (CANTAB)	1
PAL (Paired Associates Learning)	1
Judgment of Line Orientation (JLO)	5
Rey-Osterrieth Complex Figure Test	6
Wechsler Intelligence Scale for Children (WISC)	1
Working Memory Adaptive N-back Tasks	1
**Working memory**	
Behavior Rating Inventory of Executive Function (BRIEF)	1
Wechsler Intelligence Scale for Children (WISC)	1
Digit Span Forward/Backwards	1
